# Targeting delivery of simvastatin using ICAM-1 antibody-conjugated nanostructured lipid carriers for acute lung injury therapy

**DOI:** 10.1080/10717544.2016.1259369

**Published:** 2017-02-06

**Authors:** Shu-Juan Li, Xiao-Juan Wang, Jing-Bo Hu, Xu-Qi Kang, Li Chen, Xiao-Ling Xu, Xiao-Ying Ying, Sai-Ping Jiang, Yong-Zhong Du

**Affiliations:** 1 Institute of Pharmaceutics, College of Pharmaceutical Sciences, Zhejiang University, Hangzhou, PR China and; 2 Department of Pharmacy, The First Affiliated Hospital, College of Medicine, Zhejiang University, Hangzhou, PR China

**Keywords:** Lung targeting, nanostructured lipid carriers, ICAM-1, simvastatin delivery, acute lung injury

## Abstract

Acute lung injury (ALI) is a critical illness without effective therapeutic modalities currently. Recent studies indicated potential efficacy of statins for ALI, while high-dose statins was suggested to be significant for attenuating inflammation *in vivo*. Therefore, a lung-targeted drug delivery system (DDS) delivering simvastatin (SV) for ALI therapy was developed, attempting to improve the disease with a decreased dose and minimize potential adverse effects. SV-loaded nanostructured lipid carriers (SV/NLCs) with different size were prepared primarily. With particle size increasing from 143.7 nm to 337.8 nm, SV/NLCs showed increasing drug-encapsulated efficiency from 66.70% to 91.04%. Although larger SV/NLCs exhibited slower *in vitro* cellular uptake by human vascular endothelial cell line EAhy926 at initial stage, while *in vivo* distribution demonstrated higher pulmonary accumulation of the larger ones. Thus, the largest size SV/NLCs (337.8 nm) were conjugated with intercellular adhesion molecule 1 (ICAM-1) antibody (anti-ICAM/SV/NLCs) for lung-targeted study. The anti-ICAM/SV/NLCs exhibited ideal lung-targeted characteristic in lipopolysaccharide-induced ALI mice. *In vivo* i.v. administration of anti-ICAM/SV/NLCs attenuated TNF-α, IL-6 and inflammatory cells infiltration more effectively than free SV or non-targeted SV/NLCs after 48-h administration. Significant histological improvements by anti-ICAM/SV/NLCs were further revealed by H&E stain. Therefore, ICAM-1 antibody-conjugated NLCs may represent a potential lung-targeted DDS contributing to ALI therapy by statins.

## Introduction

Acute lung injury (ALI), the pre-clinical correlate of acute respiratory distress syndrome (ARDS), is a critical illness remains a health burden nowadays. Pathologically, ALI was characterized by severe inflammation with leukocytes invasion in alveoli and protein rich pulmonary edema initially, alveolar hyaline membrane formation and interstitial fibrosis subsequently, finally with various degrees of resolution (Matthay & Zemans, 2011). Despite recent therapeutic advances in supportive care and pharmacotherapies, the morbidity and mortality in ALI/ARDS still remain high (30%–40%) (Craig et al., 2011; Sadikot, 2014). The heterogeneity of etiological agents and acute pathophysiologic changes of alveolus are probably the contributory factors resulting in the disappointing outcomes (Gotts & Matthay, 2011). Therefore, reversing the multiple pathogenesis and solving the underlying causes efficiently are highlighted for ALI therapy nowadays.

Pulmonary vascular endothelium insults are recognized as the pivotal pathogenesis of ALI/ARDS (Matthay & Zemans, 2011). In addition to decreasing serum cholesterol, statins have attracted increasing attention as a therapeutic candidate for ALI nowadays, based on their endothelium-protective (endothelial cytoskeletal regulation, oxidative stress moderation) (Jacobson et al., 2004; Chen et al., 2008) and anti-inflammatory properties (down-regulation of caldesmon, up-regulation of integrin-β4) (Jacobson et al., 2004; Chen et al., 2012). The protection of endothelium contributes to the reduction of pulmonary inflammatory cells infiltration, attenuating pulmonary edema and further escalation of inflammation. The cytokines implicated in the aggravation of ALI could be reduced by simvastatin (SV) *in vivo*, which may be due to the down-regulation of MAPK signaling through up-regulating integrting-β4 (Chen et al., 2012). The protective roles of statins have been reported widely in animal models of lung injury (Jacobson et al., 2005; Muller et al., 2010; Mathew et al., 2011) and human studies (Shyamsundar et al., 2009; Craig et al., 2011). Noteworthily, high-dose statins was suggested to be significantly helpful for attenuating inflammation effectively *in vivo* (Jacobson et al., 2005; Steiner et al., 2005; Muller et al., 2010). However, excessive statins relative to human dosing regimens may induce dose-dependent systemic side effects, which manifested by the increased risk of liver toxicity and myopathy (Shyamsundar et al., 2009; Cornier & Eckel, 2015). Additionally, a prolonged treatment for improving organ dysfunction in ALI would be required relative to the anti-inflammatory efficacy achieved early by statins, which further highlighted the significance of more tolerated treatment (Craig et al., 2011). Therefore, exploring an approach to offer sufficient therapeutic cargoes in the lung with a decreased administrated dose, minimizing the potential systemic adverse effects is of great clinical significance.

Nano-drug delivery system (DDS) is an effective approach to improve drugs bio-distribution at target sites and decrease nonspecific diffusion in normal organs (Sadikot, 2014). Thereinto nanostructured lipid carriers (NLCs) have become attractive DDS due to their numerous advantages (excellent biocompatibility, feasibility of large-scale production, improvement of drug loading and storage stability) (Zhai & Zhai, 2014; Yu et al., 2016). Delivering statins using well physiologically tolerated NLCs *via* intravascular administration may benefit direct drug intervention on injured pulmonary endothelium. That contributes to the rapid onset of anti-inflammation and the improved bioavailability versus conventional oral statins delivery. Additionally, the therapeutic dose required may be decreased owing to the increased pulmonary drug delivery through passive and active targeting of DDS. Primarily, the carriers can passively accumulate in tumors or inflammation foci due to increased vascular permeability (enhanced permeation and retention effect, EPR) (Howard et al., 2014; Tang et al., 2015). While diameter-dependent nonspecific retention mediated by passive targeting has significant effect on bio-distribution of DDS (Muro et al., 2008). With respect to active targeting, a suitable target bears a vital role for targeted nanoparticles (NPs) anchoring and internalization subsequently. The endothelium is widely regarded as a significant target for DDS, in addition to the role as a “victim” needed improvement in diverse pathological conditions including inflammation (Howard et al., 2014). The endothelium encounters with circulating NPs directly in the bloodstream, providing a preferable route for pharmaceutical intervention. Based on the large surface area of pulmonary vasculature (∼25% relative to the whole endothelium in the body) and its collection of entire venous blood form right ventricle, endothelium-based lung-targeting may represent an attractive therapeutic approach for ALI nowadays. Of note, ICAM-1 is a transmembrane glycoprotein preferentially expressed on endothelial cells (ECs). The expression would be up-regulated dramatically under ALI pathological state (McClintock et al., 2008). ICAM-1 antibody-modified polystyrene particles have been widely employed in endothelium-targeted studies (Calderon et al., 2011; Bhowmick et al., 2012; Hsu et al., 2012), indicating that ICAM-1 may hold promise as an ideal endothelium-targeted determinant for pulmonary drug delivery.

In this study, SV-loaded ICAM-1 antibody-conjugated NLCs were prepared as a lung-targeted DDS for ALI therapy. SV/NLCs with different size were prepared primarily, followed by evaluating the physicochemical characteristics (diameters, drug-loading content, morphology, *in vitro* release behavior, etc.), cytotoxicity, cellular uptake and *in vivo* distribution successively. The largest size SV/NLCs (337.8 nm) in this study conductive to pulmonary drug delivery were employed to modify with ICAM-1 antibody. The lung targeting characteristics of anti-ICAM/SV/NLCs were evaluated followed. Finally, the *in vivo* pharmacodynamics after *i.v.* administration of anti-ICAM/SV/NLCs in LPS-induced ALI mice was determined.

## Materials and methods

### Materials

Monostearin was purchased from Shanghai Chemical Reagent Co., Ltd. (Shanghai, China). Polyethylene glycol monostearate (PEG_2000_-SA, M_W _= 2000) was from Tokyo Kasei Kogyo Co., Ltd. (Tokyo, Japan). Amino-terminated polyethylene glycol (NH_2_-PEG_2000_-NH_2_, M_W _= 2000) was from Yare Biotech, Inc. (Shanghai, China). SV was obtained from Zhejiang Hisun Pharmaceutical Co., Ltd. (Zhejiang, China). Medium-chain triglycerides (MCT) was gifted from Gattefosse (Saint-Priest, France). Rat anti-mouse ICAM-1 antibody (YN1/1.7.4) and mouse anti-human ICAM-1 antibody (6.5B5) were purchased from Santa Cruz Biotechnology Inc. (Santa Cruz, CA). Control IgG and ACK lysis buffer were from Beyotime Co., Ltd. (Shanghai, China). PE Rat Anti-Mouse Ly-6G and Ly-6C (Gr-1, RB6-8C5) and FITC Rat Anti-Mouse CD11b (M1/70) were purchased from BD Biosciences Inc. (San Diego, CA). TNF-α, IL-6 ELISA kits were from Boster Co., Ltd. (Wuhan, China). Near-infrared DiR fluorescent probe, Alexa Fluor® 405-conjugated Goat Anti-Mouse IgG and propidium iodide (PI) were from Life Technologies (Carlsbad, CA). Octadecylamine (ODA) was from Fluka, (Ronkonkoma, NY). Lipopolysaccharide (LPS), stearic acid (SA), N-(3-dimethylamlnopropyl)-N′-ethylcarbodiimide hydrochloride (EDC), N-hydroxysuccinimide (NHS), N,N′-disuccinimidyl carbonate (DSC), 3-(4,5-dimethylthiazol-2-yl)-2,5-diphenyltetrazolium bromide (MTT) and fluorescein isothiocyanate (FITC) were from Sigma-Aldrich (St. Louis, MO). All other solvents were of analytical or chromatographic grade.

### Preparation of blank NLCs and simvastatin-loaded NLCs

The blank NLCs were prepared by solvent diffusion method in an aqueous system. Briefly, 97.5 mg monostearin, 15 mg PEG_2000_-SA and 30 mg MCT were completely dissolved in 0.713 mL ethanol in water bath at 60 °C. Afterwards, 0.025 mL, 0.075 mL, 0.225 mL melting lipid was removed respectively and diluted to 0.25 mL by the ethanol of 60 °C. The organic phase was sequentially dispersed into 4.75 mL of 60 °C deionized water (ethanol:water = 1:19, v/v) quickly under mechanical agitation (DC-40, Hangzhou Electrical Engineering Instruments, Hangzhou, China) at 400 rpm for 30 s, followed by cooling to room temperature to obtain blank NLCs with 1 mg/mL (NLCs-1), 3 mg/mL (NLCs-2), 9 mg/mL (NLCs-3) concentration, respectively.

To prepare SV-loaded NLCs, 7.5 mg SV was added into equal lipid ingredients composition of blank NLCs followed by dissolution in 0.75 mL ethanol at 60 °C. The other procedures were the same as the mentioned above. The SV/NLCs with 1 mg/mL (SV/NLCs-1), 3 mg/mL (SV/NLCs-2), 9 mg/mL (SV/NLCs-3) concentration were prepared respectively.

### Preparation of ICAM-1 antibody-conjugated NLCs

To prepare ICAM-1 antibody-conjugated SV/NLCs, polyethylene glycol monostearate (NH_2_-PEG_2000_-SA) was employed as one ingredient. NH_2_-PEG_2000_-SA was synthesized *via* reaction between the carboxyl groups of SA and the amine groups of NH_2_-PEG_2000_-NH_2_ in the catalytic condition of EDC and NHS. Briefly, 119.8 mg EDC, 24.0 mg NHS, 35.5 mg SA were dissolved in 3 mL of anhydrous DMSO. The mixture was heated to 60 °C to activate carboxyl for 30 min under 400 rpm mechanical agitate. The reaction solution was added drop wise to 2 mL anhydrous DMSO containing 250 mg NH_2_-PEG_2000_-NH_2_ (SA:NH_2_-PEG_2000_-NH_2 _=_ _1:1, mol/mol). NH_2_-PEG_2000_-SA was obtained *via* 24 h reaction under stirring at room temperature, further dialyzed by a dialysis membrane bag (molecular cutoff = 7 kDa, Spectrum Laboratories, Laguna Hills, CA) for 48 h against distilled water. The final products were lyophilized (Labconco, FreeZone 2.5 Plus, Kansas City, MO) and stored in 4 °C for further use. The chemical structure of NH_2_-PEG_2000_-SA was confirmed by ^1^H NMR spectra and IR spectra sequentially.

ICAM-1 antibody-conjugated SV/NLCs were prepared according to our previous study (Lu et al., 2014). Briefly, 10 μL of 0.2 mg/mL NH_2_-PEG_2000_-SA solution of ethyl alcohol was added successively into the three melting lipid ingredients mentioned above, and mixed thoroughly before injecting into 4.75 mL of aqueous phase. After mechanical agitation (400 rpm, 30 s) in water bath at 60 °C and cooling to room temperature, 20 μL of 0.01 mg/mL water-DSC solution (NH_2_-PEG_2000_-SA:DSC = 1:1, mol/mol) was added into the obtained NLCs dispersion and dispersed sufficiently *via* vortex for 30 s. The reaction was proceeding at room temperature for 3 h company with slight shaking. Afterwards, 25 μg ICAM-1 antibody was added followed by slight shaking at room temperature for another 3 h. The ICAM-1 antibody-conjugated SV/NLCs with three kinds of diameters were prepared. The control IgG-conjugated 9 mg/mL SV/NLCs-3 (IgG/SV/NLCs) was prepared by the same method.

### Characterization of NLCs

#### Size, zeta potential and morphology measurements

The NLCs dispersions were diluted to 100 μg/mL (carriers concentration) with distilled water firstly. Hydrodynamic diameters, polydispersity index (PI) and zeta potential of the NLCs were determined with a Zetasizer (3000HS, Malvern Instruments Ltd, Malvern UK). The morphologies of the NLCs were viewed *via* transmission electronic microscopy with operation at 80 keV (TEM, JEM-1200EX, JEOL, Japan). The samples were pretreated with negative staining using 1% (w/v) uranyl acetate for 1 min.

#### Drug encapsulation efficiency and drug loading content

The content of SV in NLCs was determined by high-performance liquid chromatography system (HPLC), equipped with an Agilent ZORBAX 300SB-C18 column (4.6 mm × 250 mm, 5 μm) with a constant flow rate of 1 mL/min and ultraviolet detection at a wavelength of 238 nm. To test the encapsulation efficiency (EE%) and drug loading (DL%), the formulated SV/NLCs dispersions with the original carriers concentration were flocculated by adding 1 M of hydrochloric acid until the pH value was adjusted to 1.2. The SV/NLCs aggregation was separated *via* centrifugation (3K30, Sigma Labrorzentrifugen GmbH, Germany) at 20 000 rpm for 30 min. The SV concentration in the supernatant was measured. The EE% and DL% of SV in the NLCs were calculated by the following equations:

(1) $$\mathrm{EE}\;\%=\left(M_{a}-M_{s}\right)/M_{a}\times 100\%$$

(2) $$\mathrm{DL}\;\%=\left(M_{a}-M_{s}\right)/\left(M_{a}+M_{c}-M_{s}\right)\times 100\%$$

where M_s_ indicates the mass of SV in supernatant. M_a_ indicates the mass of SV added in the system. M_c_ indicates the mass of lipid added in the system.

#### *In vitro* simvastatin release from NLCs

The release test *in vitro* was measured by dialysis method. Briefly, 15 mL phosphate-buffered saline (PBS, pH 7.4) was employed as the dissolution medium at 37 °C to simulate physiological environment *in vivo*. The formulated SV/NLCs were diluted to 1 mg/mL (carriers concentration) firstly. 1 mL of the dispersions was transferred into a dialysis membrane bag (molecular cutoff = 7 kDa) and subsequently immersed in the PBS for release assay *via* an incubator shaker (60 rpm; HZ-8812S, Scientific and Educational Equipment plant, Tai Cang, China). 1 mL medium was withdrawn and 1 mL PBS was added subsequently at predetermined time points. The SV release from non-targeted SV/NLCs and ICAM-1 antibody-conjugated SV/NLCs-3 was quantified by HPLC.

### Cells culture

EAhy926 cells (EAs), a kind of cancer-endothelial hybridoma derived from fusion of human umbilical vein ECs with human lung adenocarcinoma cell line A549, was an immortalized endothelial hybrid cell line (Bouis et al., 2001). The cells were obtained from ATCC, cultured with Dulbecco’s modified Eagle’s medium (DMEM) containing 10% (v/v) fetal bovine serum (FBS) without antibiotic in the incubator at 37 °C with a humidified atmosphere containing 5% CO_2_.

### *In vitro* cytotoxicity study

Cytotoxicity was evaluated *via* MTT assay according to the manufacturer’s suggested procedures. Briefly, EAs were transferred to 96-well microtiter plates at a density of 8 × 10^3^ cells/well to grow 24 h for adherence. The cells were exposed to blank NLCs and SV/NLCs of various carriers concentrations (100 μg/mL ∼ 600 μg/mL) for 48 h afterwards. At the end of incubation, 20 μL of MTT (5 mg/mL) was added in each well for another 4 h followed by withdrawing the supernatant and adding 150 μL DMSO to dissolve the formazan *via* shaking (90 rpm, 37 °C, 30 min). The absorption was measured at 570 nm using a microplate reader (Bio-Rad, model 680, USA).

### Cellular uptake of NLCs

To evaluate cellular uptake of NLCs, octadecylamine-fluorescein isothiocyanate (ODA-FITC) was synthesized according to our previous study and employed as the fluorescent marker for the NLCs (Chai et al., 2014). To prepare fluorescently labeled SV/NLCs, ODA-FITC was added into lipid materials (ODA-FITC:lipid = 5:100, w/w) followed by preparing FITC-labeled SV/NLCs *via* solvent diffusion method as mentioned above.

EAs were transferred to 24-well plate at a density of 5 × 10^4^ cells/well to grow 24 h for adherence. Then the cells were incubated with FITC-labeled SV/NLCs (100 μγ/mL) for predetermined incubation time. Afterwards, the medium was removed and the cells were rinsed with PBS, subsequently fixed with 2% paraformaldehyde (PFA) for 10 min. The cellular fluorescence was observed by a fluorescence microscope (Leica DM4000 B; Leica, Solms, Germany). “Image J” was employed to semi-quantitatively analyze the cellular uptake level based on the obtained fluorescent images.

### Animals

All animal studies complied with the ARRIVE guidelines and were carried out under National Institutes of Health (NIH, USA) protocols, approved by the Committees of Animal Experiments at Zhejiang University in China. The male Balb/c mice (7–8 weeks) were obtained from the Zhejiang Medical Animal Centre (Hangzhou, China). Mice were housed in a climate-controlled environment with a 12/12 h light/dark cycle and provided with standard food and water.

### *In vivo* distribution study of NLCs

Near-infrared DiR (DiR:lipid = 1:100, w/w) was used instead of ODA-FITC to prepare DiR-labeled NLCs for investigating *in vivo* distribution of different size NLCs. The mice were tail-intravenously injected with 0.2 mL of DiR-labeled NLCs, which were pre-diluted to 1 mg/mL (carriers concentration). The mice were sacrificed at predetermined time points. Representative organs were collected followed by observation using the Maestro *in vivo* imaging system (Cambridge Research & Instrumentation, Inc., Woburn, MA, USA). Fluorescence intensity associated with the amount of NLCs in various organs was semi-quantified by the imaging system. The accumulation of NLCs in the organs was expressed as the average fluorescence intensity (avg signal/counts).

### *In vivo* lung targeting evaluation of ICAM-1 antibody-conjugated NLCs

The lung-targeted characteristic of targeted SV/NLCs was carried out using ICAM-1 antibody-conjugated 9 mg/mL SV/NLCs-3 (anti-ICAM/SV/NLCs). To establish murine ALI model, the male Balb/c mice (7–8 weeks) were anesthetized and challenged slowly with an intratracheal instillation of LPS (*Escherichia coli* O55:B5, 2 mg/kg) dissolved in saline (1 mg/mL) through a microinjector (Oh & Lee, 2014) for 6 h. Sham-operated mice were exposed to the same surgical procedure with an equal volume of saline. The mice were divided into the following four experimental groups: the control (healthy mice) administrated with anti-ICAM/SV/NLCs; the sham administrated with anti-ICAM/SV/NLCs; the model administrated with anti-ICAM/SV/NLCs; the model administrated with SV/NLCs-3. DiR was still employed as the fluorescent probe encapsulated in the NLCs. The mice were tail-intravenously injected with NLCs at a SV dose of 2 mg/kg (SV, 2 mg/kg) at 6 h later after the onset of LPS. At predetermined time points, the mice were euthanized followed by excising representative organs. The bio-distribution of formulated NLCs *in vivo* was observed.

To further investigate the particle size effect on NLCs bio-distribution after anti-ICAM-1 conjugation, ICAM-1 antibody-conjugated SV/NLCs with three kinds of diameters were *i.v.* injected in the model mice at the same dose with the non-targeted NLCs bio-distribution study. To investigate the effect associated with Fc region of antibody on endothelium targeting, 0.2 mL of control IgG/SV/NLCs were *i.v.* injected in the model mice simultaneously after being diluted to 1 mg/mL. The fluorescent signals in the lungs were observed after 24-h administration.

### *In vitro* cellular uptake of ICAM-1 antibody-conjugated NLCs

The EAs were stimulated with 400 ng/mL of LPS for 24 h to mimic the ECs under inflammatory pathological state. To qualitatively analyze the ICAM-1 expression *via* immunofluorescent staining, both of the quiescent EAs and activated EAs (LPS-stimulation for 24 h) were incubated with primary antibody (mouse anti-human ICAM-1 antibody) overnight at 4 °C. Afterwards, the cells were incubated with fluorescent secondary antibody (Alexa Fluor® 405-conjugated Goat Anti-Mouse IgG) for 60 min at room temperature. The nuclei were stained with PI. The cells were observed *via* a confocal microscopy (OLYMPUS FV1000) to investigate ICAM-1 expression on EAs surface after LPS stimulation.

To evaluate ICAM-1 targeting characteristic of anti-ICAM/SV/NLCs *in vitro*, the activated EAs were incubated with anti-ICAM/SV/NLCs and control IgG/SV/NLCs (20 μg/mL) simultaneously for 1 h. Meanwhile, EAs were pre-incubated with free ICAM-1 antibody (3 μγ/mL) for 1 h to block ICAM-1 epitope on the cells and then incubated with anti-ICAM/SV/NLCs. ODA-FITC was still employed as the fluorescent probe encapsulated in the formulated NLCs (ODA-FITC:lipid = 5:100, w/w). Afterwards, the EAs were harvested followed by washing with PBS *via* centrifugation. A flow cytometer (FC500MCL, Beckman Coulter) was performed to quantitatively analyze the uptake of formulated NLCs.

### *In vivo* therapeutic study

#### Experimental design

All mice were randomly divided into five groups with six mice in each group: control group, model group, SV treated group, SV/NLCs-3-treated group and anti-ICAM/SV/NLCs-treated group. The treatment groups were tail-intravenously injected with drugs at a SV dose of 2 mg/kg at 6 h later after LPS challenge. Saline was given the control and model mice simultaneously. The animals were terminated at 24 h and 48 h after onset of drug administration. Afterwards, the lungs and bronchoalveolar lavage fluid (BALF) were collected for *in vivo* pharmacodynamics study.

#### BALF collection and lung inflammation evaluation

Mouse BALF was harvested according to the procedure reported previously with a little modification (Oh & Lee, 2014). Briefly, the tracheal intubation was primarily established accompany with a ligature on the trachea by surgical lines. Ice-cold PBS (1 mL) was injected *via* the endotracheal tube and gently withdrawn repeatedly till five times. A total of 2 mL PBS was introduced and the recovery of BALF could reach to ∼80%. The recovered BALF was centrifuged at 1300 rpm for 10 min, and the supernatant was collected followed by storage at −80 °C. TNF-α and IL-6 levels in supernatant were further analyzed by ELISA assay.

To investigate the improvement of pulmonary inflammatory cells infiltration, the cell pellet harvested after centrifugation was dealt with ACK lysis buffer to remove the red blood cells according to the procedures. Afterwards, the obtained cell pellet was re-suspended by 0.5 mL of PBS. 50 μL of cell suspensions was applied to count the total number of cells through a hemocytometer. The remainder of cell suspensions was employed to assess neutrophils (PMNs) level in BALF. Briefly, cells were incubated with PE Rat Anti-Mouse Ly-6G and Ly-6C and FITC Rat Anti-Mouse CD11b (Radjabova et al., 2015) for 20 min at 4 °C according to the protocol. Then the cells were washed by PBS *via* centrifugation and finally re-suspended in 2% PFA for PMNs determination. The ratio of PMNs versus total cells in BALF was assessed *via* flow cytometer and the data were analyzed using FlowJo software. PMNs assessment was finally expressed as the concentration of PMNs in the BALF.

#### Histopathology examination

The lung tissues were excised without pulmonary lavage at 24 h after drug administration for histopathologic observation by hematoxylin and eosin (H&E) staining. Afterwards, the lungs were submerged into 10% neutral-buffered formalin for 48 h for fixation. The left lung was further dissected, dehydrated, embedded in paraffin and cut into 5 μm sections successively. The samples were stained with H&E based on the standard procedures, finally observed *via* a light microscope at 200× magnification (Nikon, ECLIPSE Ni).

### Statistics

Statistical significance between two groups was determined using Student’s *t*-tests. *p* Value < 0.05 was considered significant. All data are expressed as the mean ± SD.

## Results and discussion

### Preparation and characterization of NLCs

The particle size plays an important role in carriers distribution *in vivo* (Muro et al., 2008). Thus, non-targeted NLCs with different diameters were prepared primarily to explore the effect of particle size on pulmonary drug delivery. As shown in Table 1, blank NLCs or SV/NLCs with different carriers concentration showed significant differences in diameters. By increasing carriers concentration from 1 mg/mL to 9 mg/mL, the particle size of SV/NLCs increased from about 143.7 nm to 337.8 nm. The results suggested that NLCs of different diameters were prepared successfully through modulating lipid materials concentration in organic phase, followed by dispersing the organic phase into aqueous phase of equal volume. The SV/NLCs in this study exhibited as a DDS with relatively uniform distribution (PI, 0.170 to 0.390) and physical stability (zeta potential, −22.3 mV to −30.1 mV). Besides, Table 1 showed increasing SV-loading capacities of NLCs along with the increasing particle size, which may be due to the drug-supersaturation in aqueous phase. As the SV dosage in the NLCs was up-regulated companied with the increasing carriers concentration (fixed ratio of SV versus lipid materials), relatively fixed SV content in the dispersion phase due to drug-supersaturation may contribute to the enhanced content of SV encapsulated in the larger diameters NLCs.

**Table 1.** Physicochemical characterization of the NLCs.

Carriers	Conc. (mg/mL)	*d_*n*_* (nm)	PI	Zeta potential (mV)	EE (%)	DL (%)
NLCs-1	1	43.3 ± 11.3	0.206 ± 0.019	−19.5 ± 0.9	–	–
NLCs-2	3	85.3 ± 15.3	0.196 ± 0.028	−22.1 ± 0.5	–	–
NLCs-3	9	198.3 ± 13.3	0.193 ± 0.033	−25.4 ± 0.6	–	–
SV/NLCs-1	1	143.7 ± 2.4	0.171 ± 0.018	−23.0 ± 1.1	66.70 ± 0.88	3.29 ± 0.04
SV/NLCs-2	3	259.6 ± 9.9	0.170 ± 0.036	−22.3 ± 1.9	84.97 ± 0.43	4.15 ± 0.02
SV/NLCs-3	9	337.8 ± 25.8	0.390 ± 0.095	−30.1 ± 1.1	91.04 ± 0.87	4.43 ± 0.04
Anti-ICAM/SV/NLCs	9	354.7 ± 18.2	0.229 ± 0.051	−32.1 ± 3.2	96.78 ± 0.12	4.85 ± 0.01

The *d_n_*, PI, EE and DL represent the hydrodynamic diameters, polydispersity index, drug encapsulation efficiency and drug loading content, respectively. The data represent the mean ± SD (*n* = 3).

**Figure 1.**
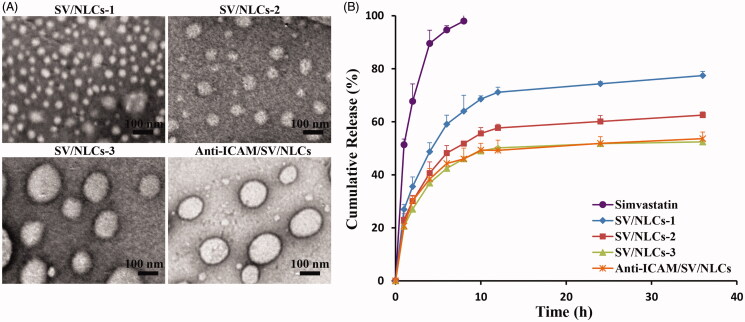
The characterization of formulated SV/NLCs. (A) Representative TEM images of formulated SV/NLCs (bar = 100 nm). (B) *In vitro* release profile of simvastatin from NLCs. The data represent the mean ± SD (*n* = 3).

Figure 1(A) showed the transmission electron microscopy (TEM) images of SV/NLCs with different carriers concentration, indicating the diameters differences and spheroidal morphologies of the SV/NLCs. Figure 1(B) revealed a diameters-correlated drug release behavior of SV/NLCs *in vitro*. The larger diameters NLCs showed slower drug release rate relative to the smaller ones at initial stage. Perhaps it is related with the smaller specific surface area of larger size NLCs (Du et al., 2010). Noteworthily, the slower drug release from larger SV/NLCs was helpful for decreasing drug leakage from carriers in the bloodstream, increasing the therapeutic agents content accumulated in the injured lung.

### The cytotoxicity and cellular uptake of NLCs

**Figure 2.**
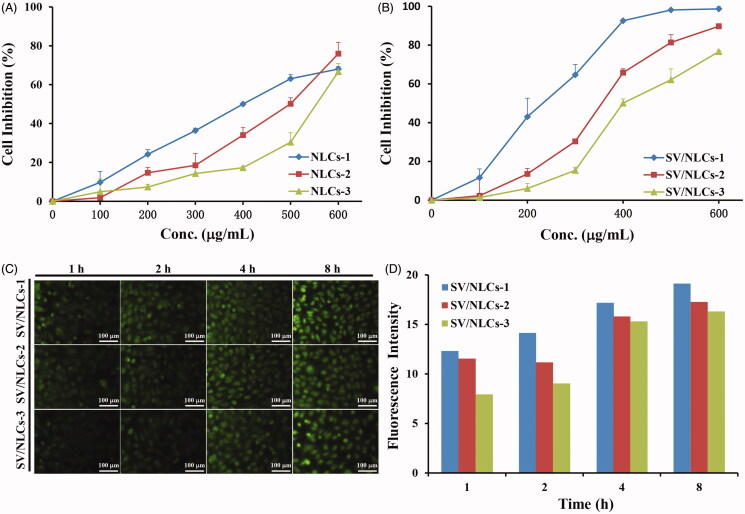
The cytotoxicity and cellular uptake of NLCs against EAs. The cells viability influence of blank NLCs (A) and SV/NLCs (B) against EAs after incubation for 48 h. (C) Cellular uptake of FITC-labeled SV/NLCs (bar = 100 μm). (D) The semi-quantitative analysis of cellular uptake by the software “Image J”. The data represent the mean ± SD (*n* = 3).

The security of NLCs to cells and cellular uptake ability *in vitro* were assessed followed. EAs have been widely used for EC research and mimicing inflammatory endothelium *in vitro* (Lanbeck et al., 2004; Kilic et al., 2006; Bhowmick et al., 2012). Given the role of pulmonary vascular endothelium as the main “victim” and therapeutic target, EAs were employed as the model cells to study the *in vitro* cells experiments in this research. MTT assay was carried out to study the security of NLCs to EAs. A dose-dependent cells inhibition rate was revealed in Figure 2(A) and (B). Besides, increasing cells inhibition rate was showed along with the NLCs diameters decreasing. It may be explained by the easier internalization of smaller size NLCs by cells relative to the larger ones. Moreover, Figure 2(A) showed the 50% cellular growth inhibitions (IC_50_) of blank NLCs in EAs were determined as about 400–550 μg/mL, suggesting that the present NLCs had low cytotoxicity on the whole (Yuan et al., 2013).

The cellular uptake test by EAs was carried out using FITC-labeled SV/NLCs. The green fluorescence signals indicated the FITC-labeled SV/NLCs inside EAs, which could be clearly observed after 1-h incubation (Figure 2C). With the extension of incubation time, gradually increasing fluorescence signals was revealed. The data demonstrated that the SV/NLCs had excellent time-dependent cellular uptake ability. That signified the nano-carriers can delivery therapeutic cargoes into cells efficiently, which is of great significance for endothelial intervention (Ansar et al., 2013). Noteworthily, the FITC-labeled SV/NLCs with smaller diameters exhibited higher fluorescence signals in EAs relative to the larger ones especially at the initial stage. The difference of fluorescence signals among different size NLCs in cells became small at long-term incubation. This may be due to the easier internalization of smaller NLCs by EAs relative to the larger ones. The semi-quantitative cellular uptake level of SV/NLCs showed the similar results with the fluorescent images (Figure 2D). Overall, the data suggested that the NLCs in this study exhibited as a promising DDS with relatively low cytotoxicity and the desired cellular internalization ability.

### *In vivo* distribution of NLCs

**Figure 3.**
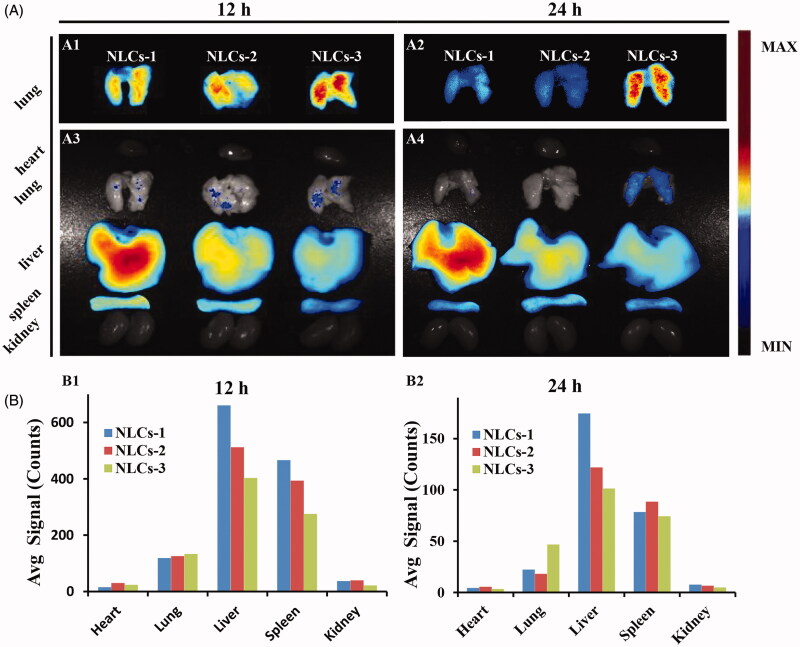
*In vivo* bio-distribution of non-targeted NLCs. (A) The fluorescence images of excised representative organs from mice. Where A1, A2 were the images of lung tissues merely observed in the same field of vision; A3, A4 were the images of the whole representative organs observed in the same field of vision. (B) The semi-quantitative fluorescence intensity in the organs, expressed as the average fluorescence intensity (avg signal/counts).

In order to investigate the effect of NLCs diameters on pulmonary drug delivery, the bio-distribution of different diameters DiR-labeled NLCs was carried out in mice. The fluorescent signals indicated the DiR-labeled NLCs in representative organs. The strongest pulmonary fluorescent signals was observed in the mice *i.v.* injected with the NLCs-3 (Figure 3), indicating that large diameters may contribute to pulmonary drug delivery. The significance of large particle size on NLCs lung-distribution after ICAM-1 antibody conjugation was further indicated in Supplementary Figure S3. On the contrary, the fluorescent signals of NLCs in livers were decreasing along with the diameters increasing. The results indicated that the larger particle size, the higher pulmonary uptake concomitant with the lower hepatic uptake. Nonspecific retention of large size NPs in pulmonary microvasculature may contribute to carriers pulmonary accumulation, which is helpful for the efficacy on injured lung and weakening the side effects on normal organs. Besides, Gentile et al. (2008) reported that larger particles might cross the endothelium more hopefully than the smaller particles due to adhering firmly to the vascular walls under flow. Therefore, SV/NLCs-3 (9 mg/mL) with the largest diameter were employed to conjugate with ICAM-1 antibody (anti-ICAM/SV/NLCs) for all subsequent study.

### Preparation, characterization and *in vivo* distribution of ICAM-1 antibody-conjugated NLCs

To prepare ICAM antibody-modified NLCs, a chemical binding method was employed to conjugate ICAM-1 antibody with the nanocarriers. NH_2_-PEG_2000_-SA was synthesized in advance as the ingredient of NLCs. The confirmation of ^1^H NMR spectra (Supplementary Figure S1) and IR spectra (Supplementary Figure S2) demonstrated the successful synthesis of NH_2_-PEG_2000_-SA. The anti-ICAM/SV/NLCs were prepared by the conjugation between the amino groups of ICAM-1 antibody and the amino group of NH_2_-PEG_2000_-SA mediated by DSC (Lu et al., 2014). The conjugation on the hydrophilic end (amino group) of NH_2_-PEG_2000_-SA contributed to exposing the ligands moiety on the NLCs surface possibly. That signified a sufficient steric flexibility for ligands anchoring on the targeted determinants in some sense.

The characterization of anti-ICAM/SV/NLCs was evaluated followed. The similar hydrodynamic diameters (337.8 nm versus 354.7 nm), DL (4.85% versus 4.43%) (Table 1) and *in vitro* drug release behavior (Figure 1B) between anti-ICAM/SV/NLCs and SV/NLCs-3 were revealed. The TEM images further confirmed the similar diameters between anti-ICAM/SV/NLCs and SV/NLCs-3, as well as the spheroidal morphologies (Figure 1A). The results indicated that ICAM-1 antibody conjugation had no significant effect on NLCs physicochemical characteristics.

**Figure 4.**
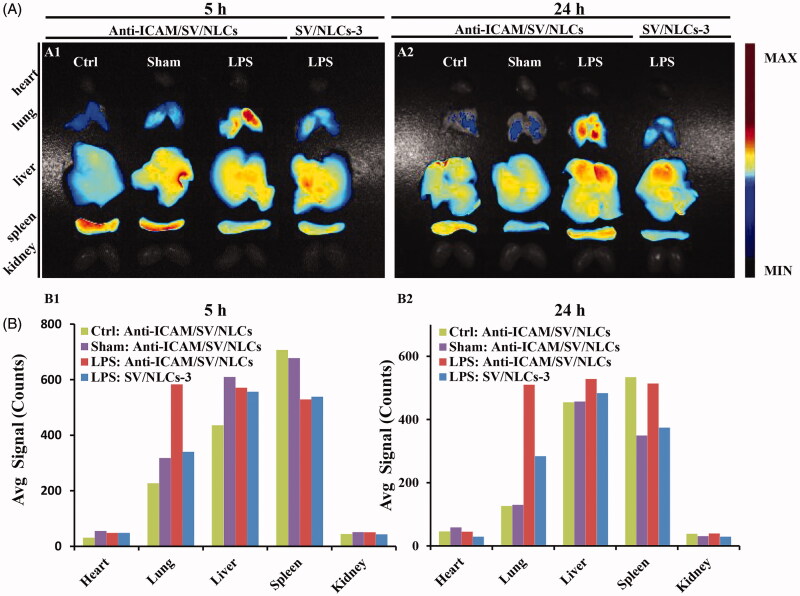
*In vivo* bio-distribution of anti-ICAM/SV/NLCs. (A) The fluorescence images of excised representative organs from mice. The column in each image sequentially showed the anti-ICAM/SV/NLCs bio-distribution in the representative organs of the healthy mice, saline-challenged mice and LPS-induced mice, from left to right. The non-targeted SV/NLCs-3 bio-distribution in the LPS-induced mice was reflected in the leftmost column. (B) The semi-quantified fluorescence intensity in the representative organs, expressed as the average fluorescence intensity (avg signal/counts).

With respect to the lung targeted characteristic of anti-ICAM/SV/NLCs *in vivo*, the influence of ICAM-1 determinant expression on carriers accumulation in lung was investigated. The bio-distribution of anti-ICAM/SV/NLCs was evaluated in healthy mice, sham-operated mice, and LPS-challenged mice known with increased ICAM-1 expression (Calderon et al., 2011). Figure 4 showed the strongest fluorescent signals in the lung of LPS-challenged mice (e.g. 583.3 in the model versus 229.4 in the healthy versus 318.2 in the sham after 5-h administration, avg signal/counts). Slightly stronger signals was observed in the lungs of the sham relative to the healthy, which was likely due to the up-regulated ICAM-1 expression or the EPR effect in inflammation foci (Howard et al., 2014), given the potential inflammation triggered by surgery. The results demonstrated the positively correlated impact of targeted determinant expression on carriers binding *in vivo*. Besides, the low pulmonary signals of anti-ICAM/SV/NLCs in the healthy and sham-operated mice suggested that the carriers may provide selectivity toward injured pulmonary endothelium with high ICAM-1 expression. That may contribute to the reduced carriers binding on healthy endothelium with basal ICAM-1 expression, weakening the potential side effects after *i.v.* administration.

The bio-distribution of anti-ICAM/SV/NLCs and their non-targeted counterparts in LPS-challenged mice was compared simultaneously. More stronger pulmonary signals of anti-ICAM/SV/NLCs was revealed relative to non-targeted SV/NLCs-3 in Figure 4. Besides, Supplementary Figure S3 showed higher signals of anti-ICAM/SV/NLCs versus control IgG/SV/NLCs in the lung, suggesting that lung-targeting characteristic of anti-ICAM/SV/NLCs was not attributed to the nonspecific binding of Fc region and Fc receptors. These results demonstrated the multivalent interactions of anti-ICAM/SV/NLCs with over-expressed ICAM-1 on pathological pulmonary endothelium may facilitate carriers anchoring efficiently *in vivo*, contributing to the enhanced pulmonary drugs accumulation. With respect to the LPS-challenged mice administrated with anti-ICAM/SV/NLCs for 5 h, the strongest fluorescent signals was revealed in the lung (583.3, avg signal/counts) relative to other represent organs (49.5 in heart, 570.9 in liver, 529.1 in spleen, 51.5 in kidney, avg signal/counts; Figure 4(B1)). The results indicated that anti-ICAM/SV/NLCs may delivery SV efficiently to injured lung. That indicated the effective improvement of ALI may be realized with a decreased administrated dose of statins. Collectively, lung targeting characteristic of anti-ICAM/SV/NLCs was suggested here.

### Cellular uptake study of ICAM-1 antibody-conjugated NLCs

**Figure 5.**
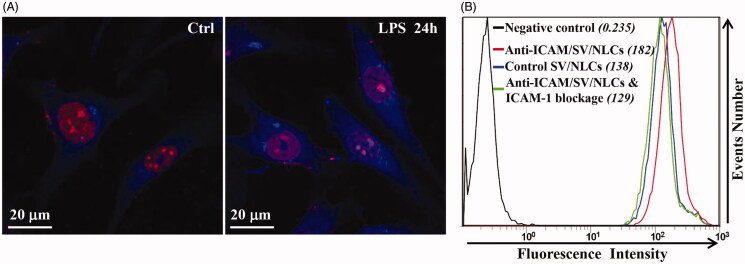
The cellular uptake of anti-ICAM/SV/NLCs. (A) The representative immunofluorescence images revealed the expression of ICAM-1 on EAs (bar = 20 μm). (B) The quantitative analysis of ICAM-1 targeting characteristic of anti-ICAM/SV/NLCs *in vitro* by a flow cytometer.

To further evaluate the ICAM-1 targeting specificity *in vitro*, cellular uptake by activated EAs was carried out using anti-ICAM/SV/NLCs and their non-targeted counterparts. Meanwhile ICAM-1 blockage experiment was carried out to further investigate the endocytosis pathway of anti-ICAM/SV/NLCs. Immunofluorescence images showed increased fluorescence signals of LPS-induced EAs compared with resting EAs, which proved the up-regulated expression of ICAM-1 on EAs surface after LPS stimulation (Figure 5A). The results suggested the establishment of pathological endothelium with over-expressed ICAM-1 *in vitro* (Calderon et al., 2011). Figure 5(B) exhibited obvious difference of mean fluorescence intensity (MFIs) between the cells incubated with anti-ICAM/SV/NLCs (182) and control IgG/SV/NLCs (138), which indicated higher uptake of anti-ICAM/SV/NLCs compared with their non-targeted counterparts. Besides, the cellular uptake of anti-ICAM/SV/NLCs was decreased after ICAM-1 blockage (129). The data suggested the widely recognized CAM-mediated endocytosis of anti-ICAM-NPs (Bhowmick et al., 2012; Hsu et al., 2012) may facilitate the internalization of anti-ICAM/SV/NLCs. Therefore, the ICAM-1 targeting specificity of anti-ICAM/SV/NLCs instead of nonspecific binding *in vitro* was suggested. The interactions between anti-ICAM/SV/NLCs and ICAM-1 epitopes may contribute to the subsequent internalization. It is of great significance to enhance drugs accumulation in the pathological endothelium and improve endothelial intervention.

### *In vivo* anti-inflammatory efficacy assessments

**Figure 6.**
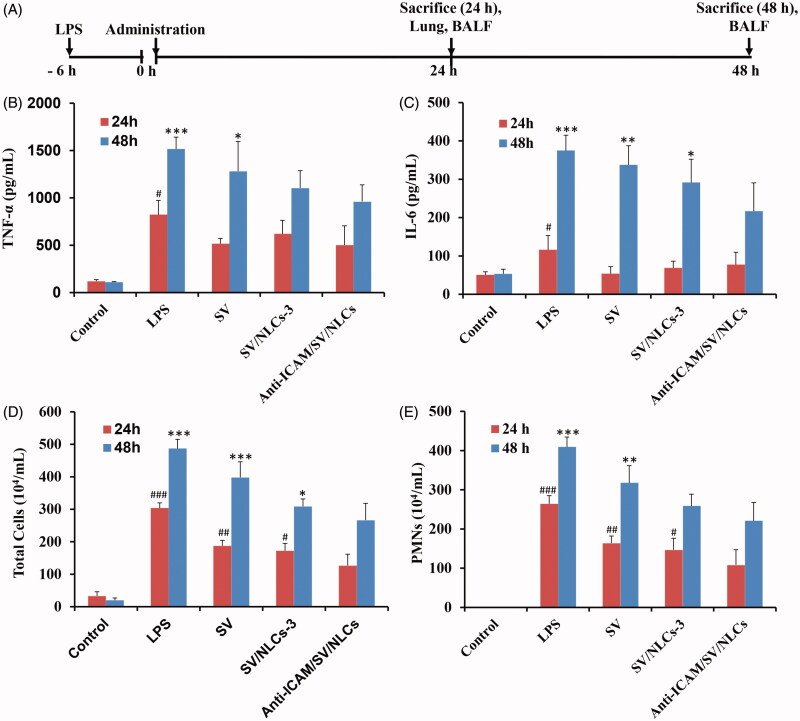
*In vivo* anti-inflammatory assessments. (A) Treatment schedule. (B) TNF-α level. (C) IL-6 level. (D) Total cells counts. (E) Neutrophils counts. #*p* < 0.05, ##*p* < 0.01, ###*p* < 0.001 indicated the indices of corresponding groups compared with anti-ICAM/SV/NLCs treatment group after 24-h administration. *p < 0.05, **p < 0.01, ***p < 0.001 indicated the indices of corresponding groups compared with anti-ICAM/SV/NLCs treatment group after 48-h administration. The data represent the mean ± SD (*n* = 6).

The therapeutic efficacy of anti-ICAM/SV/NLCs was finally evaluated in a murine model of ALI. The experimental scheme was illustrated in Figure 6(A). The disorder of inflammatory mediators is a crucial characteristic of ALI, which further amplifies inflammatory cascades and leads to severe injury (Huo et al., 2013). Thus two pivotal cytokines (TNF-α and IL-6) levels in BALF were assessed to confirm the anti-inflammatory efficacy of anti-ICAM/SV/NLCs. As shown in Figure 6(B) and (C), both of the inflammatory cytokines pronouncedly increased after LPS challenge (e.g. ∼6.8-fold versus the control for TNF-α, ∼2.3-fold versus the control for IL-6 at 24 h). Various degrees attenuation of inflammatory cytokines in treatment groups was revealed. Noteworthily, the administration of anti-ICAM/SV/NLCs exhibited a significant attenuation in inflammatory cytokines compared with SV/NLCs-3 or free SV after 48-h administration. The therapeutic advantage of anti-ICAM/SV/NLCs relative to other treatments in this study was possibly due to the following reasons: (i) the ideal lung-distribution ability. More amount of SV may be delivered to the injured lung by anti-ICAM/SV/NLCs compared with other treatments. The higher dose of SV intervened with pulmonary endothelium contributed to the more effective inhibition in inflammation; (ii) prolonged release of SV from the NLCs. It is well known that only the released drug molecules from carriers can exhibit efficient therapy activity for diseases. As the *in vitro* release profile revealed (Figure 1B), anti-ICAM/SV/NLCs presented a prolonged release of SV during 36 h. That may explain why anti-ICAM/SV/NLCs showed the more significant attenuation of inflammation relative to free SV at a long-term administration (48 h). Meanwhile, the sustained release may also lead to reduced drugs leakage in the bloodstream, decreasing the potential system side effects while increasing the SV content delivered into the pathological pulmonary endothelium.

Inflammatory cells infiltration in pulmonary interstitium and alveolar is another important symptom of ALI, leading to pulmonary edema and further escalation of inflammation (Matthay & Zemans, 2011). Therefore, the pulmonary cells infiltration was tested simultaneously to evaluate ALI-protection efficacy of anti-ICAM/SV/NLCs. Wherein the PMNs were determined by the specific identification of their typical epitopes of Gr-1 and CD11b (PE-Gr1+, FITC-CD11b+) (Radjabova et al., 2015). The total cells number severely increased in the BALF after LPS-stimulation, manifesting as PMNs mainly (87.0% and 84.0% of the total cells at 24 h and 48 h, respectively; Figure 6D and E). Relative to the improvement of inflammatory cytokines, anti-ICAM/SV/NLCs showed significant inflammatory cells attenuation versus other treatments not only at 48-h administration but also at 24 h. While the efficacy between anti-ICAM/SV/NLCs and free SV treatment groups still exhibited more obvious statistical significance after 48-h administration (*p* < 0.001, total cells) compared with 24-h administration (*p* < 0.01, total cells), indicating the effect of prolonged release. The results demonstrated the positive activities of anti-ICAM/SV/NLCs in reducing lung inflammatory cells numbers, owing to the protective effects on vascular barrier and ideal lung targeting characteristics.

**Figure 7.**
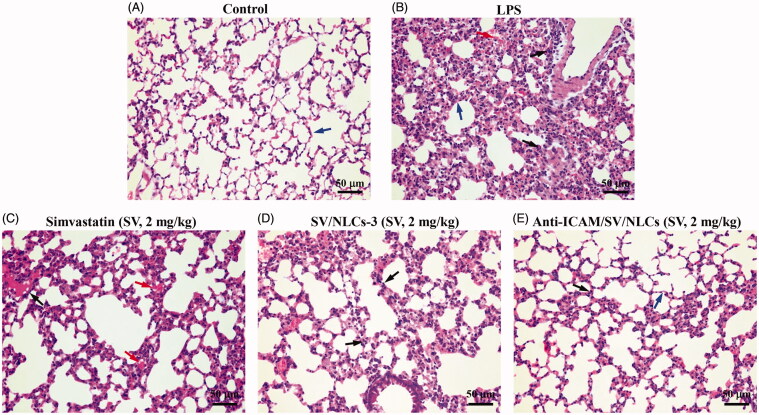
Histopathological examination. Lung section of (A) control mice, (B) LPS-induced model mice, (C) free simvastatin-treated mice, (D) non-targeted SV/NLCs-3-treated mice, (E) anti-ICAM/SV/NLCs-treated mice. The blue arrows indicate alveolar wall. The red arrows depict the alveolar wall hyperemia. The black arrows indicate the neutrophils infiltration (bar = 50 μm).

Moreover, the improvements of pulmonary tissue was observed by H&E staining. The lungs of model mice showed diverse pathological changes including diffuse alveolar damage, alveolar walls thickening, dramatical leukocytes infiltration, congestion relative to the control (Figure 7A and B). The significant histological improvements were observed after treatments (Figure 7C–E). The anti-ICAM/SV/NLCs showed the significant amelioration of alveolar wall thickening and inflammatory cells infiltration relative to other treatments. Overall, the data suggested that anti-ICAM/SV/NLCs exhibited considerable ALI improvement capacity. The prolonged efficacy of anti-ICAM/SV/NLCs compared with free SV may conductive to reduced administration frequency after appropriate optimization, improving safety and patient compliance.

## Conclusions

In the present study, ICAM-1 antibody-conjugated SV-loaded NLCs were prepared and their potential for murine ALI improvement was evaluated. The formulated NLCs could encapsulate the SV effectively and exhibited prolonged drug release for 36 h. Relatively low cytotoxicity to EAs and excellent ECs uptake ability of the NLCs was revealed. Besides, anti-ICAM/SV/NLCs exhibited ideal lung-targeted characteristic in LPS-induced ALI mice, indicating increased drug accumulation in injured lung. The *i.v.* administration of anti-ICAM/SV/NLCs exhibited considerable and prolonged efficacy in the model mice. The results suggested that, anti-ICAM-1 modified NLCs may represent a potential lung-targeted DDS contributing to the therapy of ALI by statins.

## Supplementary Material

Supporting_Information.docx

## Notes

**Supplementary materials available online.**
