# Supplementary material for: Targeting delivery of simvastatin using ICAM-1 antibody-conjugated nanostructured lipid carriers for acute lung injury therapy
Source: Drug Deliv. 2017 Feb 6;24(1):402–13. doi: 10.1080/10717544.2016.1259369 (PMC8248938; doi:10.1080/10717544.2016.1259369)
Supplement: Supporting_Information.docx [file IDRD_A_1259369_SM4942.docx]

**Supporting information**


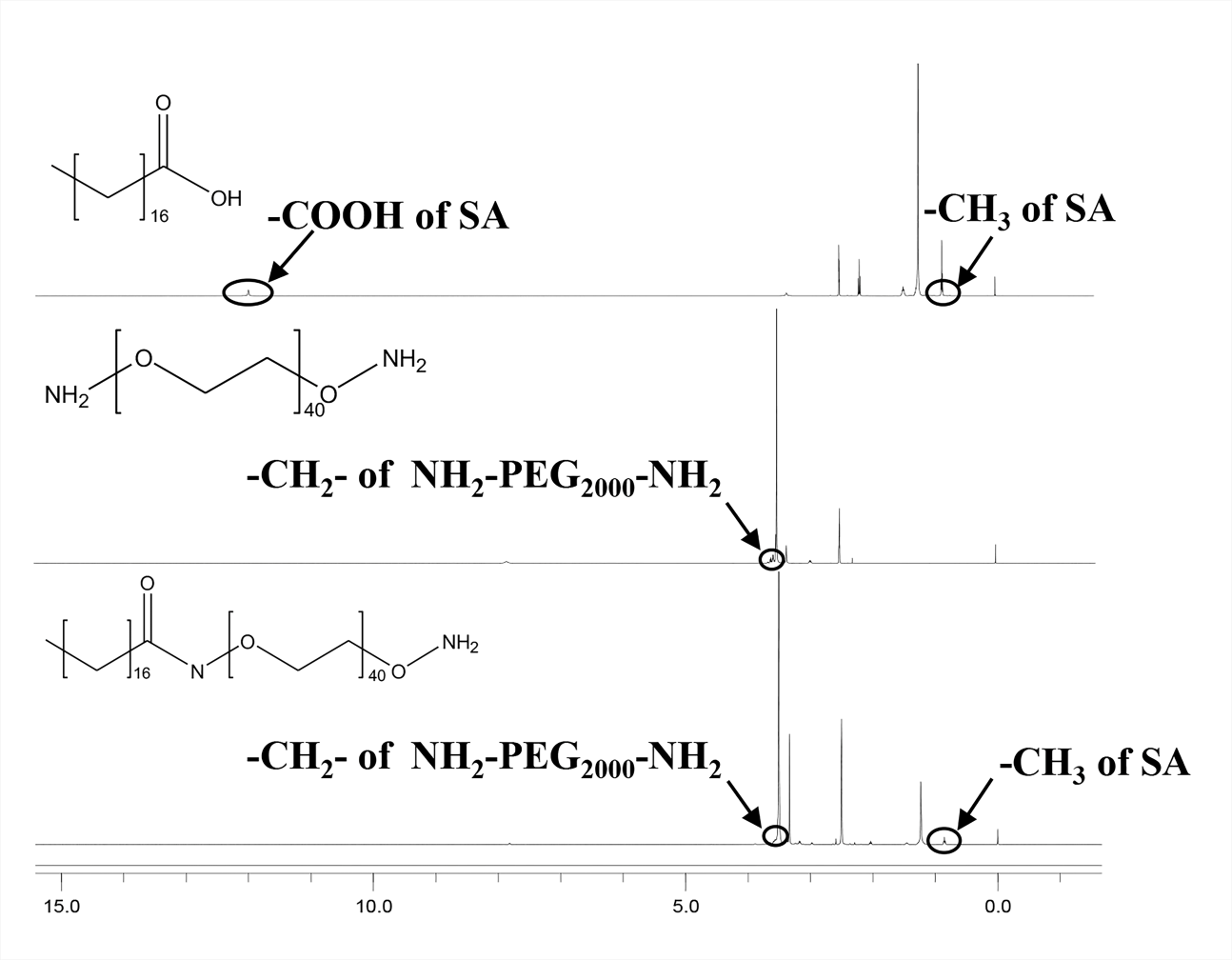


**Figure S1.** ^1^H NMR spectra of stearic acid (SA), NH_2_-PEG_2000_-NH_2_ and NH_2_-PEG_2000_-SA. The proton peak of -CH_3_ (0.96 ppm) of SA and -CH_2_- (3.63 ppm) of NH_2_-PEG_2000_-NH_2_ were detectable in ^1^H NMR spectra of NH_2_-PEG_2000_-SA, while the proton peak of carboxyl of SA (12.00 ppm) disappeared.


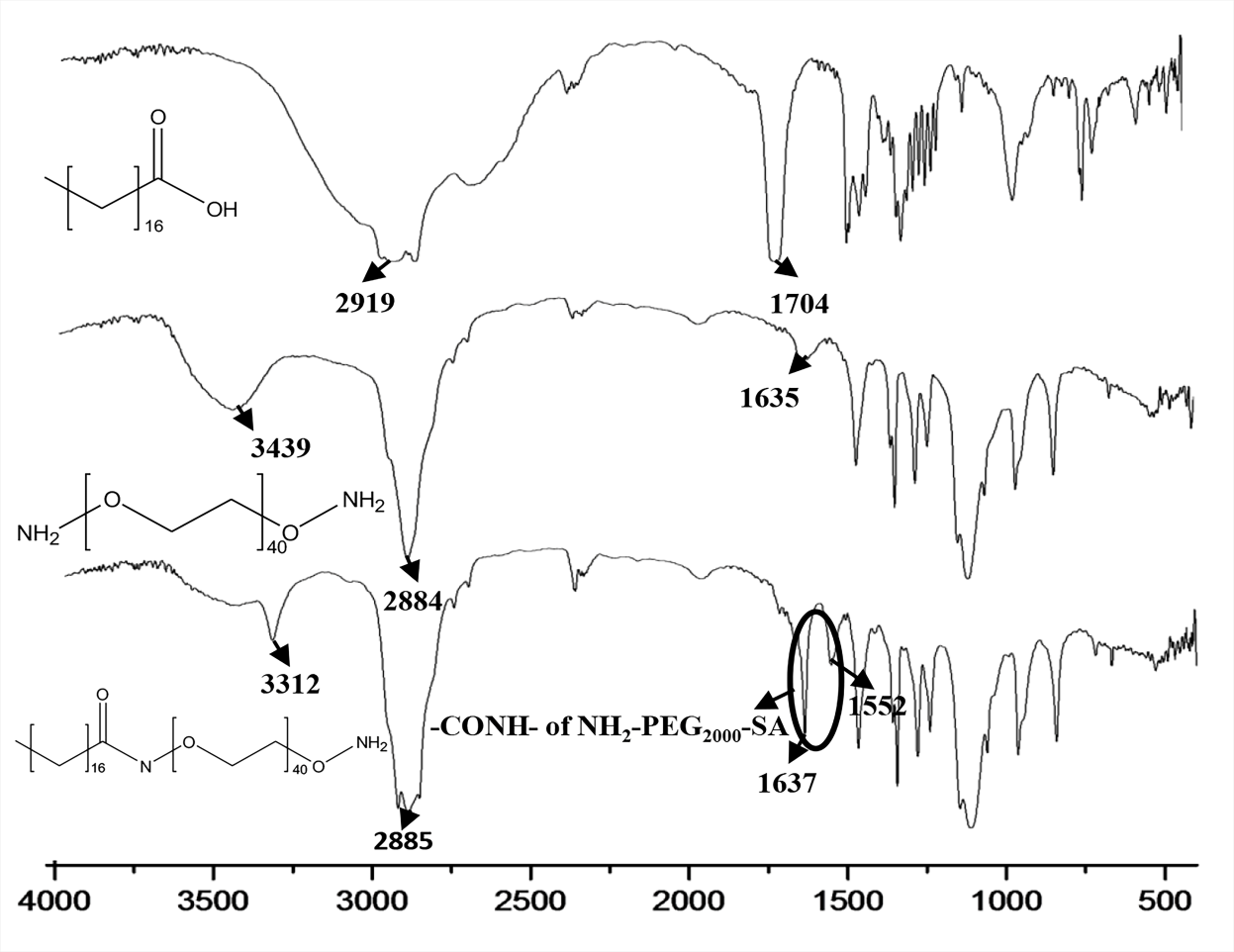


**Figure S2.** IR spectra of SA, NH_2_-PEG_2000_-NH_2_ and NH_2_-PEG_2000_-SA. The signal assigned to the -CONH- (1637 cm^-1^, 1552 cm^-1^) was detected in IR spectra of NH_2_-PEG_2000_-SA.

**
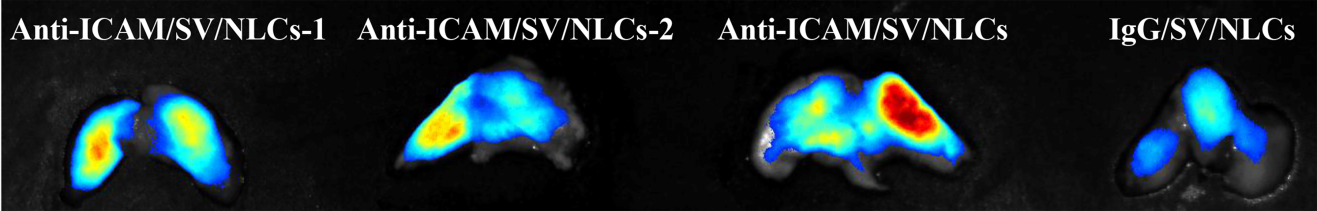
**

**Figure S3.** The pulmonary distribution of ICAM-1 antibody conjugated SV/NLCs with three kinds of diameters and control IgG/SV/NLCs. The anti-ICAM/SV/NLCs-1, anti-ICAM/SV/NLCs-2 and anti-ICAM/SV/NLCs represent ICAM-1 antibody conjugated SV/NLCs with the concentration of 1 mg/mL, 3 mg/mL, 9 mg/mL respectively. The IgG/SV/NLCs represent the control IgG conjugated 9mg/mL SV/NLCs-3.
